# The Fontan procedure is no longer the last operation

**DOI:** 10.1093/icvts/ivab369

**Published:** 2022-01-21

**Authors:** Alyssia Venna, Yves d’Udekem

**Affiliations:** Division of Cardiac Surgery, Children’s National Hospital, Washington, DC, USA

**Keywords:** Fontan, Reoperation

How many times have we not said that the Fontan procedure is the last in a series of procedures for palliation of single-ventricle conditions? We now understand that the majority of these patients will likely require a reoperation within their lifetime. Larger studies, such as those done by the Australia and New Zealand Fontan Registry and Children’s Hospital of Philadelphia, have found the reintervention rate to be as high as 50–75% within the first 3 decades of life [1, 2]. While we do realize that these patients have an increasing need for reoperation, we are still uncertain of the best timing at which to offer this surgery. In this perspective, the article in this issue by Nakayama *et al.* [3] brings some light to this question. In their retrospective work, the procedures presented are somewhat different than those we will face in the future. Nakayama *et al.* present a large series of Fontan conversions with remarkably good result. From a general consensus, Fontan conversion has become an obsolete procedure because the majority of patients who had atriopulmonary connections have either been reoperated or they are in such a condition that they are no longer a candidate mainly because of their decreased ventricular function. Clearly, we have performed, and will continue to perform, a large number of pacemaker procedures.

We are now facing an increasing number of single-ventricle patients requiring atrioventricular valve and semilunar valve reoperation. Some have predicted that a large number of patients with pulmonary root as the main semilunar valve conduit supporting the systemic circulation will eventually dilate [4]. It is likely that we will encounter a larger number of these procedures in the future. There is an epidemic of patients with atrioventricular valve regurgitation. Interestingly, this work, like others, points to the fact that replacement of these valves may be a viable option in this population [5, 6].

The most striking feature of this study is the very low mortality of these patients, and more importantly, the fact that this mortality has decreased in recent times. The cardiologists following these patients are rightly reluctant to offer an operation to patients who are often asymptomatic, because the expected high mortality of the reoperation procedure, the low rate of success and the lack of data concerning the benefits of this reoperation. The work of Nakayama *et al.*, with their low mortality and excellent 10-year survival may influence us in these decisions. Finally, they importantly demonstrated that operating on these patients at an earlier stage and before they reach failure decreases the risks of these operations. This goes along with the recent PEACH score publication by Constantine *et al.* [7] who showed that, for similar lesions, adults with congenital heart disease should have a reoperation far earlier than their counterparts with biventricular circulation and acquired heart disease.

This article by Nakayama *et al.* is adding insight into how well we are performing reoperations following Fontan. It is refreshing to know that although there are higher rates of reoperation in patients with Fontan circulation, they are being performed successfully with lower rates of mortality.

**Conflict of interest:** Yves d’Udekem is a consultant for Actelion. The other author reports no conflict of interest.
